# Unveiling the Conservation Biogeography of a Data-Deficient Endangered Bird Species under Climate Change

**DOI:** 10.1371/journal.pone.0084529

**Published:** 2014-01-03

**Authors:** Junhua Hu, Yang Liu

**Affiliations:** 1 Key Laboratory of Mountain Ecological Restoration and Bioresource Utilization & Ecological Restoration Biodiversity Conservation Key Laboratory of Sichuan Province, Chengdu Institute of Biology, Chinese Academy of Sciences, Chengdu, China; 2 State Key Laboratory of Biocontrol and School of Life Sciences, Sun Yat-sen University, Guangzhou, China; Università degli Studi di Napoli Federico II, Italy

## Abstract

It remains a challenge to identify the geographical patterns and underlying environmental associations of species with unique ecological niches and distinct behaviors. This in turn hinders our understanding of the ecology as well as effective conservation management of threatened species. The white-eared night heron (*Gorsachius magnificus*) is a non-migratory nocturnal bird species that has a patchy distribution in the mountainous forests of East Asia. It is currently categorized as “Endangered” on the IUCN Red List, primarily due to its restricted range and fragmented habitat. To improve our knowledge of the biogeography and conservation of this species, we modeled the geographical pattern of its suitable habitat and evaluated the potential impacts of climate change using ecological niche modeling with a maximum entropy approach implemented in Maxent. Our results indicated that the amount of suitable habitat in all of East Asia was about 130 000 km^2^, which can be spatially subdivided into several mountain ranges in southern and southwestern China and northern Vietnam. The extent of suitable habitat range may shrink by more than 35% under a predicted changing climate when assuming the most pessimistic condition of dispersal, while some more suitable habitat would be available if the heron could disperse unrestrainedly. The significant future changes in habitat suitability suggested for *Gorsachius magnificus* urge caution in any downgrading of Red List status that may be considered. Our results also discern potentially suitable areas for future survey efforts on new populations. Overall, this study demonstrates that ecological niche modeling offers an important tool for evaluating the habitat suitability and potential impacts of climate change on an enigmatic and endangered species based on limited presence data.

## Introduction

The application of biogeography principles, theories, and analyses in conservation biology, has contributed substantially to the conservation of global biodiversity [1], [2]. One of the preliminary tasks of conservation biogeography is to explore the geographic patterns of species to inform conservation and management actions [2]. Such efforts can significantly improve our knowledge of the evolutionary history and conservation status of species of concern, allowing effective conservation management [3]. Efforts have been made through field surveys, distribution sampling and modeling to better understand geographical patterns of biodiversity.

Ecological niche modeling (ENM) is widely used to address issues in biogeography, global change ecology, and conservation biology [4]–[6]. ENM utilizes species presence data and associated ecological variables, e.g. physical and environmental conditions, to map areas of suitable habitat for the species in question [5]. Improving the efficiency of the ENMs, i.e. identifying area with the highest conservation value, establishment of protected areas, implementation of suitable conservation measures and determining the potential impacts of predicted future climate change on species' range shift, is a critical point for the conservation biology [7]–[10].

Some animal species occupy habitats that are difficult to access, and/or have peculiar behavioral patterns, which are difficult to be detected. For example, it is challenging to collect presence data of species that inhabit remote alpine mountains, tropical forests, or polar regions [11]–[13]. Current distribution patterns reflect only a snapshot in the evolutionary trajectory of a species whose distribution may shift due to intrinsic and extrinsic factors, such as range expansion [14] and environmental changes [15]. There is increasing evidence that shifts in species distribution are increasing with the ever-changing climate [15]–[17]. Understanding species biogeography can also be hindered by peculiar ecology and distinct behaviors that are not conducive to the collection of their presence/abundance data. For example, data regarding secretive and nocturnal species, such as carnivores, bats and owls tend to be more difficult to obtain than those of more active and diurnal species [12], [18]. All these difficulties pose challenges to the understanding and application of conservation biogeography.

The white-eared night heron *Gorsachius magnificus* (Ogilvie Grant, 1899; hereafter WENH), under the family Ardeidae, is a medium-sized heron endemic to East Asia [19]. This species is distributed in the subtropical and tropical moist lowland forests of southern and southwestern China and northern Vietnam. It’s nocturnal habit and secretive behavior makes it a little-known bird species [20], [21]. Population status is poorly documented and its distribution is known to be scattered and spatially patchy [22], as indicated by limited specimens and field sighting records [23]–[25]. Due to the fragmented range and small population size (with an estimate of <1 000 adult individuals), it is currently listed as “Endangered” in the IUCN Red List [22]. Habitat requirements are poorly understood. What is known is that it relies on forest for breeding and water bodies for foraging [20]–[22]. Recent studies on distribution demonstrated that it may be more widespread in southern and southwestern China than previously thought [20], [24], with an extent of occurrence approximately 2.5×10^6^ km^2^ [26]. The core range is in South China, a densely populated region with a long history of human development, posing considerable pressures to conservation. This highlights the urgent priority for improvement in the conservation biogeography of this endangered, mysterious species to inform management and conservation planning [27].

Several recent presence records indicate new localities of WENH likely owing to increased awareness and survey efforts [24], [26], but a complete picture of its range and potential suitable habitats is still not well established. This lack of information may hinder effective conservation efforts, particularly under the context of habitat degradation and climate change [28]. Thus, this study aims to provide new insights into the biogeography and conservation management of this globally threatened species. To achieve this goal, we used the ENM [5] to characterize the potential suitable distribution range of WENH, and model the potential impacts of predicted future climate change on the species’ range. We also discussed the implications of our results for conservation and management actions.

## Materials and Methods

### Species Presence Data

We obtained georeferenced data of all known presences of WENH from published literature [21]–[26], [29]–[31] and other unpublished sources that we considered reliable sightings (Table S1). All presence data were treated equally without consideration of the population size at each locality. The presence data were double-checked using spreadsheets and geographic information system (GIS) to detect duplicates and possible georeferencing errors. This yielded a final compilation of 36 presence records. We tested for the presence of spatial autocorrelation for these records using the average nearest neighbor index in spatial statistics tools of ArcGIS 9.2 (ESRI, Redland, USA). The spatial pattern of presence data was shown to be neither clustered nor dispersed based on the Manhattan distance (nearest neighbor ratio = 1.04, Z = 0.50, *p = *0.62).

### Environmental Variables

We initially compiled 31 environmental variables (describing bioclimatic features, habitats, anthropogenic impacts and etc.), for modeling the potential distribution range of WENH (Table S2). Nineteen bioclimatic variables were obtained from the WorldClim 1.4 database [32]. We obtained water body data from the Global Lakes and Wetlands Database [33], and the NDVI data (normalized difference vegetation index, as the average of values for 12 months over a 18-year period from 1982 to 2000; http://edit.csic.es/Soil-Vegetation-LandCover.html). Since distance to water is perhaps the most important ecological factor for waterbirds, based on the water body data, we classified each grid-cell's distance to water by creating a Euclidean distance-based raster in spatial statistics tools of ArcGIS 9.2 (ESRI, Redland, USA). To represent soil-water balance and soil properties, we included growing degree days, net primary productivity, soil moisture, soil organic carbon, and soil pH from the Center for Sustainability and the Global Environment (http://www.sage.wisc.edu/atlas/index.php), as well as, annual actual evapotranspiration (*AET*_anu_), annual aridity index, and annual potential evapotranspiration from the Consortium for Spatial Information (http://www.cgiar-csi.org). To incorporate anthropogenic impacts on WENH, we used the human footprint index (HF), which is an estimate of human influence based on human settlements, land transformation, accessibility and infrastructure data [34]. We obtained the compound topographic index (CTI, commonly referred to as the wetness index) that was representative of the topography variability from the United States Geological Survey’s Hydro1K dataset (http://edcdaac.usgs.gov/gtopo30/hydro/).

The inclusion of all environmental variables may create uncertainty in the ENM predictions [35], [36]. Over-fitting could happen if there are too many variables, especially for the small sample size of presence records [37], [38]. Due to the high levels of correlations between many environmental variables and the need for variables to be as proximal as possible, we filtered the initial variable set based on the results of Pearson’s correlation test and jackknife analysis. We executed correlation tests across all pair-wise combinations of variables. For highly correlated variable pairs (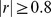 ), we retained the variable that gave a higher value in the regularized gain and/or the percent contribution to the Maxent model [39]. Consequently, ten environmental variables were identified: mean monthly temperature range (*T*_ran_), isothermality (*T*_iso_), mean temperature of the warmest quarter (*T*_war_), annual precipitation (*Prec*_anu_), precipitation of the driest month (*Prec*_dry_), *AET*_anu_, CTI, HF, distance to water and NDVI. All variables were resampled to a resolution of 2.5 arc-min using a bilinear interpolation function which is considered to be more realistic than the simpler nearest-neighbor method [39].

### Bioclimatic Variables of Future Climate Projections

Given the uncertainty in future climate projections, we used two CO_2_ emission scenarios (A2a and B2a) from the Fourth Intergovernmental Panel on Climate Change (IPCC) Special Report [40]. A2a has medium to high CO_2_ emissions while B2a has low to medium emissions. Data were derived from three general circulation models: CCCMA [41], CSIRO [42] and HADCM3 [43]. The five selected bioclimatic variables were extracted under different climate change scenarios to make projections into the year 2050. Because no scenarios were available for the future development of non-climatic variables (i.e. *AET*_anu_, CTI, HF, distance to water and NDVI) [44], these variables were assumed constant. Therefore, our predictions represented a conservative estimate of the combined impacts from environmental change on the distribution range of WENH.

To define climatic variation, we extracted the values of bioclimatic variables at species presence records under both current and future scenarios using spatial analysis tools in ArcGIS 9.2 (ESRI, Redland, USA). The divergence between current and future climate scenarios associated with *T*_ran_, *T*_iso_, *T*_war_ and *Prec_anu_* was examined by independent samples *t*-tests. We used Kolmogorov–Smirnov tests to check for normality of data and transformed data to meet normality assumptions when necessary. The parameter *T*_iso_ was log transformed and *T*_ran_ was arctan transformed. We tested if *Prec_dry_* differed between the current and future scenarios using a nonparametric Mann-Whitney *U*-test. Finally, we extracted the value of bioclimatic variables within projected suitable ranges under different scenarios to examine the climatic variation between current and future predicted ranges. The divergence associated with bioclimatic variables between current and future scenarios was examined by a nonparametric Kruskal-Wallis test. These analyses were conducted in SPSS 16 (SPSS Inc., Chicago, 2005).

### Ecological Niche Modeling

**Figure 1:**
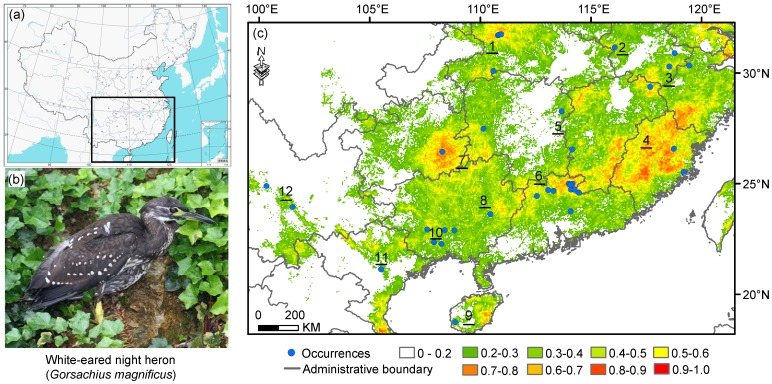
Study area and predicted habitat suitability for the endangered white-eared night heron *Gorsachius magnificus*. The suitability is displayed in logistic value and ranges from 0 (lowest suitability) to 1 (highest suitability). Blue circles indicate known occurrence records. The Arabic numerals indicate locations of the significant mountain ranges mentioned in this study: 1, Shennongjia Mt.; 2, Dabie Mt.; 3, Huangshan-Tianmu Mt.; 4, Yandang-Wuyi-Daiyun Mt.; 5, Mufu-Jiuling-Luoxiao Mt.; 6, Nanling Mt.; 7, Wuling-Xuefeng-Miaoling Mt.; 8, Dayaoshan Mt.; 9, Limuling-Wuzhishan Mt.; 10, Shiwan'dashan Mt.; 11, Ban Thi & Xuan Lac; 12, Ailao Mt.

We constructed ENM for WENH with Maxent version 3.3.3k [39]. Maxent is a machine learning method specifically designed for presence-only data. It has been shown to have good predictive performance across various applications [8]–[10], [45], [46]. Maxent uses environmental variables to predict environmental suitability for a particular species by assessing different combinations of variables and their interactions using the maximum entropy principle [39]. The complexity of Maxent models can be controlled through choice of feature classes and regularization parameters [47]. We mainly used default settings in this study. We ran models with 10 bootstrap replicates, and assessed model performance using the average AUC (area under the receiver operating curve) score (mean ± SD) by randomly assigning the presence records as training and test datasets (80 and 20%, respectively). Additionally, referred to the procedure introduced by Raes and ter Steege (2007) [48], we applied a null-model approach to test whether our established ENMs of WENH significantly differed from what would be expected by chance. The AUC value of the real ENM was determined using all presence records and a null-model was generated by randomly drawing collection localities from the geographical area for which ENMs were developed. We used logistic output format which was easily interpretable with logistic suitability values ranging from 0 (lowest suitability) to 1 (highest suitability) [45]. We defined the extent of the study for the Maxent model as covering the entire known range of WENH (Fig. 1) [24], [26]. We ensured only one presence per grid-cell at the resolution of 2.5-arc-min when we performed models.

The consensus-based ensemble-forecasting approach that combines a number of alternative models to forecast species distribution is considered to provide robust projections [49], by combining different realizations of possible statuses of the real distribution represented by different models. Because different ENMs provide considerably different results [50]–[52], which is the very source of the largest variation in the projections of climate change impacts among the main sources of uncertainty examined (e.g. initial datasets, ENMs, general circulation models and gas emission scenarios) [53], we used the bootstrap replicates from Maxent as proxies for different single-models in the consensus methods [49]. We summarized the output predictions from three general circulation models under the two emission scenarios by calculating the mean suitability within each grid-cell in ArcGIS 9.2 (ESRI, Redland, USA) [52].

### Spatial Analysis of the Impacts of Climate Change

We assessed the changes of potential distribution under predicted future climate change using the following approaches. Because the dispersal ability of WENH is unknown, we considered two scenarios of dispersal ability on different ends of the spectrum, i.e. null spread (no dispersal ability) and full spread (unlimited dispersal ability) [44]. A species will only persist in overlapping areas between current and future projected ranges under the null spread scenario, while it can colonize all suitable ranges under the full dispersal scenario. Suitable range was defined on the Boolean (presence/absence) map that was transformed from continuous suitability outputs by corresponding thresholds or “cut-offs” [35], [54]. We estimated the suitability for WENH, under both the current and future climate conditions for each grid-cell based on the average training presence threshold of the output maps. To assess suitable range variation at the pixel level, we quantified the potential range loss (RL) by pixel and related this to the current suitable range (CR) by pixel. Under the full spread assumption, the percentage of range gained (RG) was assessed by the same procedure; we estimated the percentage of predicted range change (RC) [55] using 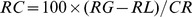 and turnover (RT) using 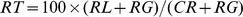 . To support conservation decision making, we also illustrated the projected stable range, range loss, and range gain.

To examine the spatial trend of WENH’s range change in response to climate change, we calculated the mean, minimum, and maximum suitability values, elevations, latitudes, and longitudes of the grid-cells of suitable range for both current and the corresponding future scenarios [56], using spatial analysis tools in ArcGIS 9.2 (ESRI, Redland, USA). We then categorized and summed the total area of suitable range (number of grid cells) per latitudinal band, to investigate the latitudinal pattern of the resulting gain or loss in suitable range under projected climate change.

Finally, to remedy uncertainties associated with the choice of thresholds to convert logistic model outputs into binary estimates of presence and absence [35], [54], we additionally calculated the change in habitat suitability as the difference of the suitability between current and future climate scenarios without choosing any type of threshold [57].

## Results

### Model Performance, Explanatory Variables and Predicted Distribution

The AUC values revealed that our model can provide reasonable discrimination (AUC_training_ = 0.886±0.016; AUC_test_ = 0.817±0.047). Additionally, with the 95% confidence interval upper limit AUC value (0.786) from the frequency histogram of the null-model, the ENM of WENH had an accuracy that was significantly higher than expected by chance alone (*p*<0.01; Fig. S1). Environmental variables that contributed the most to modeling the potential distribution were *Prec*_dry_, NDVI and *T*_iso_. In contrast, *T*_ran_, and *Prec_anu_* made only small contributions to model development (Table S3).

Most areas of the study region were predicted to have low suitability for WENH (Fig. 1). 90.7% of grid cells had a logistic suitability value <0.5. Moreover, 5.4% (*c.* 1.9×10^5^ km^2^), 2.9% (*c.* 1.0×10^5^ km^2^), and 0.9% (*c.* 3.2×10^4^ km^2^) of habitat were classified to have a suitability value of 0.5–0.6, 0.6–0.7 and 0.7–0.8 respectively. Only 0.07% (*c.* 2.5×10^3^ km^2^) of habitat was with a logistic value of >0.8. Additionally, 1.3×10^5^ km^2^ of the output map can be considered as the suitable distribution range of WENH.

Model outputs clearly identified five areas as containing highly suitable habitat for WENH: eastern to southwestern China, western Hubei province, southern Anhui province to northeastern Jiangxi province, Hainan, and northeastern Vietnam (Fig. 1). The predicted distribution range generally matches with available presence records. However, both Yunnan and Hunan provinces in China were projected to have low suitability, although both had known presences of the species.

**Figure 2:**
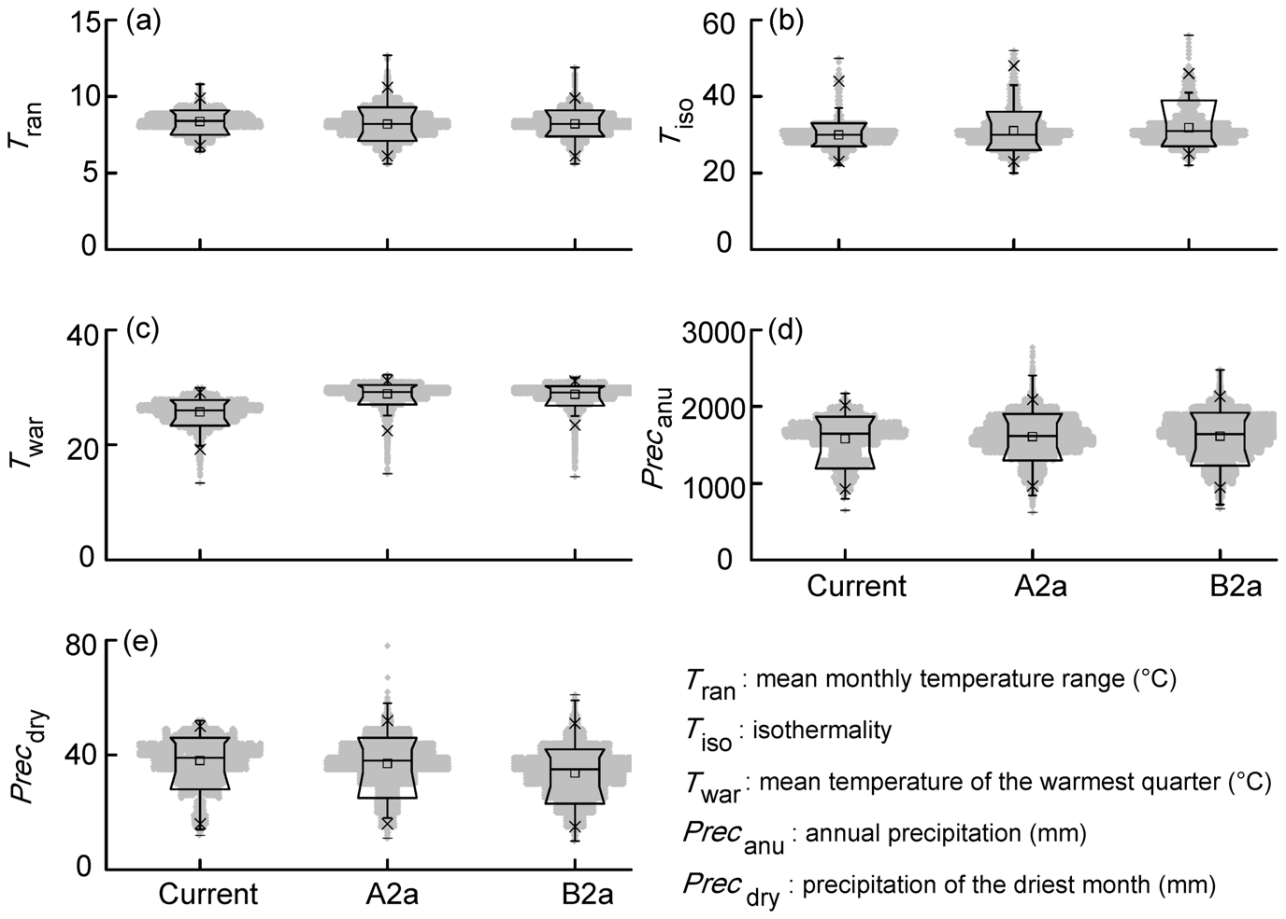
Comparison values of bioclimatic variables in projected suitable range between current and future climate scenarios. The black solid horizontal line represents the median, the square symbol represents the mean, edges of box are quartiles, whiskers are the 1th and 99th percentiles and black short lines are minimum and maximum.

When comparing current value of bioclimatic variables on known presences to those values under the climate change scenarios, current *T*_war_ was significantly lower than future *T*_war_ (*t* = −4.03, *p*<0.0001) while other comparisons were not significantly different (*T*_ran_: *t = *1.09, *p = *0.28; *T*_iso_: *t = *0.02, *p = *0.99; *Prec_anu_*: *t* = −1.09, *p = *0.28; *Prec_dry_*: *U = *3578, *p = *0.44; Fig. S2). For values of bioclimatic variables within suitable range, *T*_iso_, *T*_war_ and *Prec*_anu_ were lower at present than in future scenarios (all *p*<0.001, Kruskal-Wallis tests), while *T*_ran_ and *Prec_dry_* were higher at present than in future scenarios (both *p*<0.001, Kruskal-Wallis tests; Fig. 2).

### Potential Effects of Climate Change

**Figure 3:**
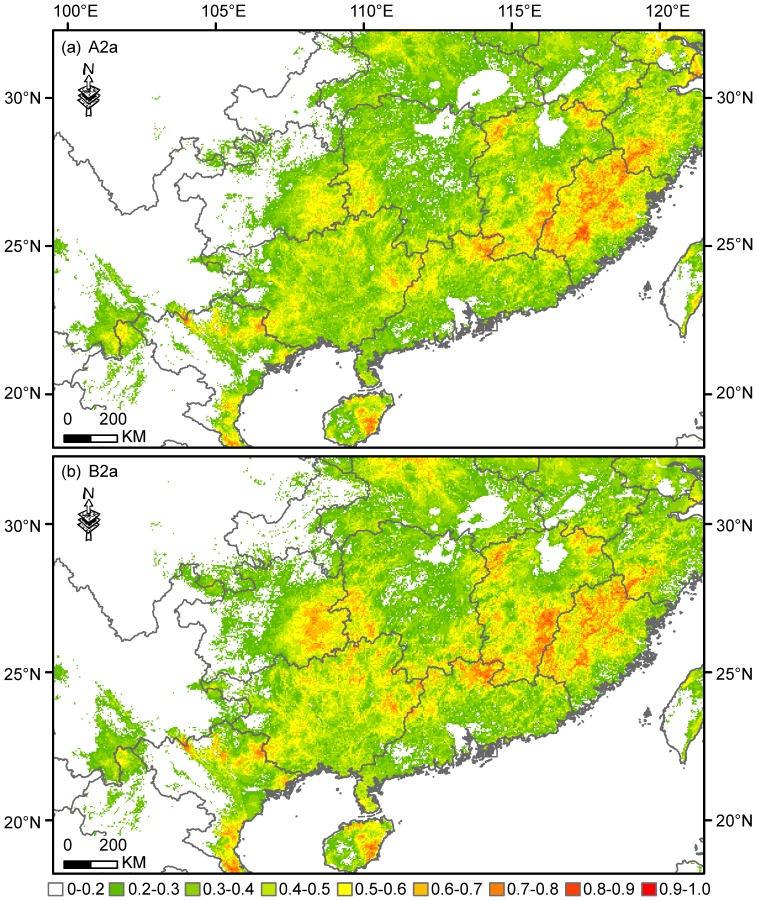
Predicted habitat suitability for *Gorsachius magnificus* under the projected climate scenarios in 2050. These predictions are obtained with an ensemble-forecast approach across the three general circulation models CCCMA, CSIRO and HADCM3 of the climate scenarios under the two storylines (A2a and B2a).

The projected effects of climate change on the suitable range of WENH were identifiable (Fig. 3). Under the climate change scenario A2a, 6.9% (*c.* 2.39×10^5^ km^2^), 3.0% (*c.* 1.03×10^5^ km^2^), and 0.9% (*c.* 3.20×10^4^ km^2^) of grid-cells were classified to have a logistic suitability value of 0.5–0.6, 0.6–0.7 and 0.7–0.8, respectively. The number of grid cells with logistic prediction of >0.8 (0.06%, *c.* 2.35×10^3^ km^2^) was slightly less than that at present. Under the scenario B2a, 7.9% (*c.* 2.75×10^5^ km^2^), 4.0% (*c.* 1.38×10^5^ km^2^), and 1.3% (*c.* 4.36×10^4^ km^2^) of grid cells were classified to have a logistic value of 0.5–0.6, 0.6–0.7 and 0.7–0.8, respectively. The grid cells with logistic prediction >0.8 (0.09%, *c.* 3.18×10^3^ km^2^) were about five quarters of that at present.

Under the climate change scenarios A2a and B2a, the percentage of range loss for WENH was respectively predicted to be 35% and 36%. The percentage of range gain was 36% and 73%, under A2a and B2a, respectively. These combined to produce a percentage of range turnovers of 52% and 63% under A2a and B2a, respectively. Under the full dispersal assumption, a range increase of 1% and 37% was projected under A2a and B2a, respectively. The average extent of areas that herons were predicted to occur would increase to 1.32×10^5^ km^2^ and 1.80×10^5^ km^2^, respectively.

**Figure 4:**
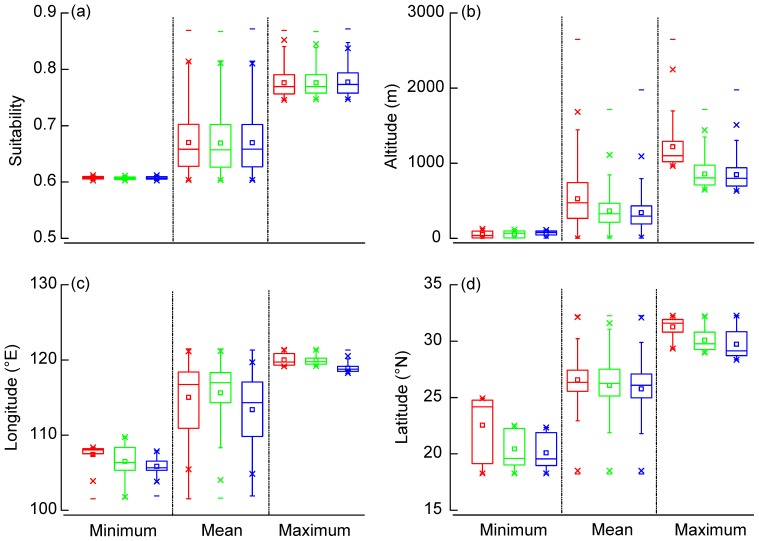
Discrepancy of suitability, altitude, longitude and latitude for predicted distribution between current and future scenarios. The minimum, mean, and maximum of suitability (a), altitude (b), longitude (c), and latitude (d) for predicted distribution of *Gorsachius magnificus* are exhibited. Red, green and blue represent current, A2a and B2a scenarios, respectively.

Suitable range in current condition was predicted to not become less or more suitable for WENH under climate change, with no significant change in mean logistic prediction and maximum logistic prediction. However, the area of minimum prediction was predicted to decrease under A2a (Z = 3.86, *p*<0.001; Fig. 4a). The elevational distribution was predicted to decrease in upper limit (by 361 m under A2a, Z = 21.50, *p*<0.001; by 370 m under B2a, Z = 24.43, *p*<0.001) and in mean value (by 162 m under A2a, Z = 24.42, *p*<0.001; by 184 m under B2a, Z = 32.77, *p*<0.001; Fig. 4b) while it was predicted to increase in lower limit (by 9 m under A2a, Z = 3.62, *p*<0.01; by 18 m under B2a, Z = 7.46, *p*<0.001). Overall, our models predicted longitudinal distribution shifts, with a mean westward shift of 1.65° under B2a, a maximum shift of 0.01° under A2a, and of 1.10° under B2a, and a minimum shift of 0.92° under A2a, and of 1.58° under B2a (Fig. 4c). Suitable areas were predicted to shift southward in mean latitude (by 0.49° under A2a, and by 0.80° under B2a), in maximum latitude (by 1.18° under A2a, and by 1.54° under B2a) and in minimum latitude (by 2.11° under A2a, and by 2.45° under B2a; Fig. 4d).

**Figure 5:**
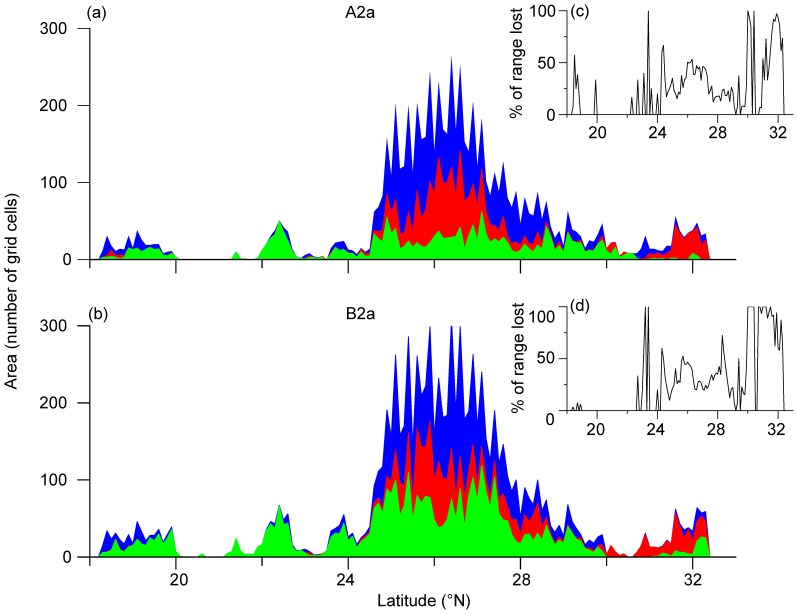
Impacts of climate change on the habitat suitability along the latitude. Panels a and b represent the projected impacts for *Gorsachius magnificus* under two future climate scenarios (A2a and B2a; blue, current suitable ranges projected to be stable; red, suitable ranges projected to be lost; and green, the suitable ranges projected to be newly gained), respectively. The panels c and d represent the percentage of range lost. The predicted suitability is estimated based on the average training presence threshold.

**Figure 6:**
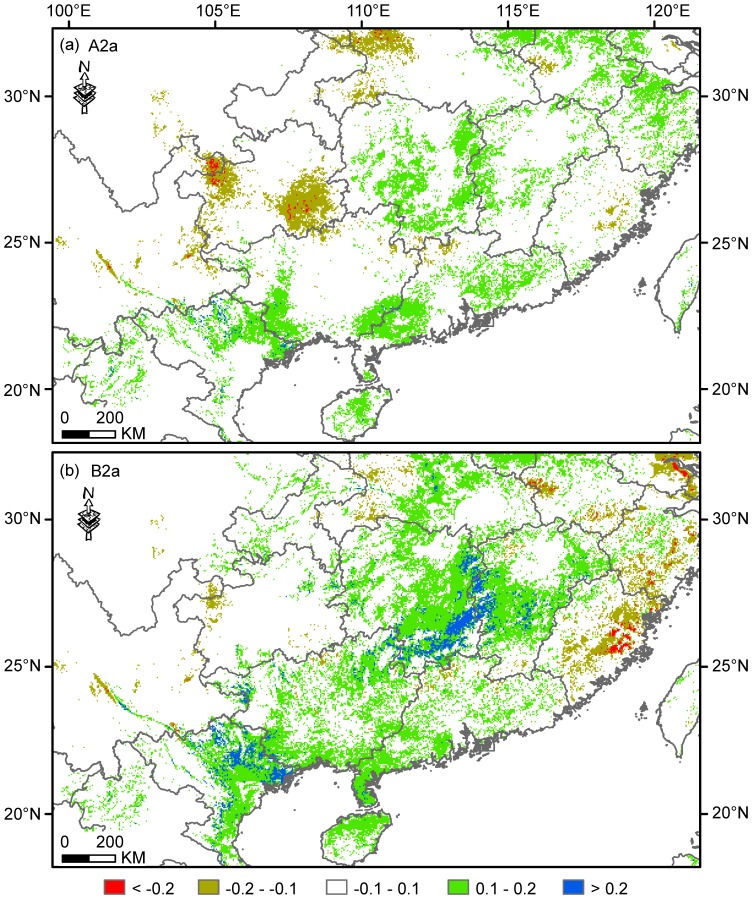
Disagreement of projected habitat suitability between current and future scenarios. The disagreement is calculated as the divergence of habitat suitability of *Gorsachius magnificus* between current and future climate conditions under the A2a (panel a) and B2a (panel b) climate scenarios. The negative values represent that the habitat is less suitable in future than in current, while the positive values represent the habitat is more suitable in future than in current.

Along latitudinal gradient, compared with the current distribution, suitable range loss was predicted to mainly happen at mid to high latitudes. This trend of range loss along latitudinal gradient was consistent with that of range projected to be stable, regardless of the emissions scenario used (A2a or B2a; Fig. 5a & b). Range gains in the future were predicted to scatteredly occur along latitudinal gradient under the A2a scenario, whereas this trend was consistent with that of range loss and stable under the B2a scenario. The percentage of predicted range loss along latitude was shown to be low below 20° N and was relatively high after a gap ranging 20° to 22° N (Fig. 5c & d). Spatially explicit comparisons between current and future projected ranges showed that more than 75% of grid cells had a low divergence range −0.1–0.1 in logistic suitability prediction (Fig. 6). Additionally, under A2a, 3.7% (1.27×10^5^ km^2^) and 0.12% (4.03×10^3^ km^2^) of current range were predicted to decrease by 0.1–0.2 and >0.2 in logistic suitability prediction, respectively. In contrast 11.6% (4.00×10^5^ km^2^) and 0.19% (6.53×10^3^ km^2^) of current range were predicted to increase by 0.1–0.2 and >0.2, respectively. Under scenario B2a, 3.5% (1.20×10^5^ km^2^) and 0.24% (8.18×10^3^ km^2^) of current range were predicted to decrease with 0.1–0.2 and >0.2, respectively. In contrast, 18.6% (6.43×10^5^ km^2^) and 1.99% (6.89×10^4^ km^2^) of current range were predicted to increase by 0.1–0.2 and >0.2, respectively. These spatially explicit comparisons identified high divergences in Guangxi, Guangdong, Hubei, Anhui and Fujian provinces of China and northeastern Vietnam under either scenario A2a or B2a (Fig. 6).

## Discussion

Our study shows that suitable habitat for the WENH is scattered in the mountainous areas of southern and southwestern China and northern Vietnam. The current presence records of WENH largely fall in this range, which is much congruent with the range defined by BirdLife International [35], [46], [58]. Although the estimated suitable range spans a wide geographical area, it is generally patchy. Despite the lack of field records, we found substantial areas of presumably suitable habitats in the east part of this range (i.e. Fujian and Guizhou), which is a candidate region to search for new populations. We further demonstrated that the current suitable range would decrease under the projected climate scenarios if WENH cannot disperse unrestrainedly. Altogether, these results provide new insights into the biogeographic pattern of the most enigmatic heron species in the world and are useful information guiding effective conservation management.

Although the availability of presence records is a considerable limitation for the application of ENMs for threatened species, Maxent is recognized to be accurate and stable across all tested sample-size categories [35], [46], [58]. Additionally, because sampling bias exists in our presence records Maxent provides a solution to both omission and commission errors using both presence records and randomly selected background samples [39]. Acceptable values of AUC and the validation with the null-model here confirmed the effectiveness of using Maxent for the implementation of ENM for WENH. The results of ENM have been suggested to be only a spatial projection of realized niche estimated from the input presences and environmental predictors but not necessarily reflected the current occupied range [59]. Thus it is crucial to consider the actual habitat requirement of a species and involve relevant and operable environmental layers in ENM. In this study, besides the bioclimatic layers, we actually considered the relevant non-climatic layers, especially the layer of distance to water that is important to a waterbird species. Therefore, the suitable distribution projected here represents the habitat requirement of WENH reasonably.

### Biogeographic Pattern

Our results showed that the WENH is distributed between 18–32° N and 100–120°E, with about 130 000 km^2^ of potentially suitable habitat. This area is only about 5% of the extent calculated by He et al. [26]. The range covers 11 provinces in southern and southwestern China and northern Vietnam but habitats of high suitability (i.e. with a logistic prediction value of >0.8) are restricted and scattered within the mountain chains of southern China. These areas include several mountain ranges, namely the Huangshan-Tianmu Mountains, Yandang-Wuyi-Daiyun Mountains, Mufu-Jiuling-Luoxiao Mountains, Wuling-Xuefeng-Miaoling Mountains, Nanling Mountains, and Shiwan’dashan Mountains (Fig. 1). The two peripheral mountains, the Shennongjia and Dabie Mountains, represent the northern limit of this species [60]. The southwestern limits of WENH are around the Ailao Mountains and Ban Thi & Xuan Lac of Vietnam, both of which contributed recent records of the species [21], [26]. Historical records from the Limuling-Wuzhishan Mountains in Hainan Province constitute the southernmost distribution limit of WENH is isolated from the rest of the species’ range.

Apart from regions with only historical records, e.g. Hainan and the northernmost Dabieshan, recent sightings or evidence of breeding colonies have been reported in other mountain ranges mentioned above [20], [21], [24], [26]. These records suggest that independent and self-sustaining subpopulations may inhabit these regions. He et al. estimated 11 subpopulations corresponding to the major mountain chains within the heron’s range based on review of field records [25]. However, it should be noted that the spatial division of these subpopulations was tentative without supporting genetic information. While the WENH is mainly distributed in the subtropical mountainous forests in southern and southwest China, this region harbors several other endemic montane bird species with pronounced phylogeographic structures, such as *Tragopan caboti* [61] and *Stachyridopsis ruficeps* [62]. We therefore consider that the postulated subpopulations may correspond to distinct evolutionary units. Genetic analyses are necessary to assess genetic diversity, as well as to obtain sound understandings of the population connectivity between mountain ranges [63].

The recent rediscovered Fujian subpopulation is only the second record from this province since a historical breeding colony in northern Fujian [26]. Models predict that suitable habitat is projected to occur along the Yandang-Wuyi-Daiyun Mountain chains in southern Zhejiang and central Fujian. This projected distribution may be habitats for breeding populations that have not yet had field confirmation. Another region with relatively large areas of suitable habitat that may warrant field surveys are the Miaoling Mountains between southeastern Guizhou and northern Guangxi. Our results suggest that these mentioned regions require further field survey efforts as they may contain existing undiscovered subpopulations.

### Effects of Climate Change

Although climate change was suggested to pose less effect in the tropics and subtropics than temperate zones or polar regions, increasing frequencies of extreme weathers (e.g. severe droughts, large fires, storms, and flooding) are threatening the ecosystems of the tropics and subtropics [64]. Few studies have evaluated the impacts of climate change on the terrestrial biota in South and East Asia (but see Zhou et al. [65]). Due to the current limited distribution, climate change may substantially affect WENH by reducing its current suitable range. We attempted to illustrate patterns of distribution under climate change using an up-to-date ENM, because bird species generally exhibit predictable responses to changes in temperature and precipitation [55], [56], [66]. As expected, the projected distribution under future climate scenarios suggests that current suitable habitat will become more limited with a considerable proportion of current suitable habitat predicted to be lost. Under the unlimited dispersal assumption, projected appropriable colonization locations in the future are similar to that of lost in the climate change scenario A2a but are twice as much as that of lost in the scenario B2a. Consequently, distribution would be likely to decrease in the future if this species cannot disperse to the new appropriable colonization locations. This situation is most likely to occur due to the high intensity of human activities and habitat fragmentation through much of range of WENH [22], [26]. Although predictions from our study carry considerable uncertainties, the predicted distribution changes under relatively conservative scenarios suggest considerable challenges to, and demands for, conservation actions. Specifically, climate change is shown to cause increased mortality and decreased growth rates in large trees in a subtropical monsoon evergreen broad-leaved forest in southern China [65]. This change in forest community can induce habitat degradation in the region, which might influence the breeding site of WENH. Moreover, habitat fragmentation and habitat loss (mainly led by illegal logging) are considered key threats to this heron through much of its range [22], [26]. Thus, any climate change effect on suitable habitat will compound the challenge of preserving suitable habitat adequately.

Climate change is suggested to alter the distribution of WENH, and may threaten the species’ population viability. Population-level impact of climate change has been found in many species in many parts of the world [67]–[70]. Climate is recognized to be a dominant environmental factor dictating the distribution of species [5], [17]. It is therefore critical to understand the effects of climate change on species' distribution [70]. Under a changing climate, some species will benefit by extending ranges into currently unsuitable areas, however, many species exhibit negative feedbacks that will reduce their distribution range and/or accelerate extinction rates [44], [55], [56], [67], [68]. Species with a small geographic range tends to be more vulnerable to climate change than more widely distributed species [56], [67], [68]. More importantly, the primary effect of the involvement of climate extremes, which is a correction of local over- and under-prediction, can improve models of species range limits under future conditions [71]. In line with this statement, our results show that the habitat change under the scenario B2a on is larger than that of the A2a. This seems intuitively reverse because the scenario B2a described less severe climate change situation [72]. In our case, however, precipitation of the driest month (*Prec*_dry_) contributed the most to the ecological niche modeling of WENH among environmental variables. Due to the value of *Prec*_dry_ in the B2a is lower than that in the A2a within the projected suitable range (Fig. 2), the effects of the scenario B2a on the potential distribution of WENH are most likely to be larger than that of the A2a. These results highlight the importance of incorporating climate change, especially climate extreme, into the habitat conservation planning of endangered species.

### Implications for Species Conservation

The WENH is currently listed as an “Endangered” species in the IUCN Red Data list under the criteria of EN C2 (i) for its small and fragmented population and pressures from hunting and deforestation in current habitats [22]. Though updated presence data and observations in southern China [24], [25], [73] have been expand our knowledge on the distribution and ecology of WENH, these few data seem to be weak to inform conservation efforts given the wider distribution of this species in the region. In stead, by taking biophysical and environmental conditions into account and using robust spatially explicit modeling techniques, our study quantified habitat suitability of WENH in its range for the first time. The results of this study have several implications for the conservation of WENH. First, our ENM reveals the existence of several patchy suitable habitats WENH in its range in southern China (Fig. 1c). This appears to support the assessment of fragmented population by IUCN. Second, apart from several known key regions that require special attention in research and conservation management, we identify southern Guizhou and northern Fujian as the regions demanding survey efforts aiming for searching new subpopulations. Third, most suitable habitats of WENH are in integral montane subtropical forests though, some records are located in less-suitable habitats (i.e. locations 10 and 12 in Fig. 1c), which may be exposed to more habitat loss and human disturbance in the future. New nature reserves or protected areas should consider to be established there. Further, we examined the range of WENH under climate change scenarios and found its current range may shrink considerably under future climate change while new suitable habitat may be available if WENH can disperse unrestrainedly (Figs. 4–6). The predicated significant future changes in habitat suitability suggested for this species urge caution in any downgrading of Red List status that may be considered.

In conclusion, our study presents a robust, spatially explicit model of the conservation biogeography of an enigmatic and endangered bird species. We demonstrate the utility of ecological niche modeling in predicting potential distribution and future threats posed by climate change. Future research efforts should be targeted on population size estimation, surveys in locations of potential distribution, and conservation genetic analyses. Combined with the knowledge of large-scale biogeography, it is necessary to conduct research on fine-scale habitat use. This will enables us to understand the patchy distribution and habitat choice of WENH. Apart from research, management actions should be focused on protecting known habitats and nesting sites, as well as raising awareness to reduce habitat degradation and human disturbance. Collectively, these efforts will not only provide critical information to enable a complete understanding of the distribution and population of the WENH, but will aid in implementing effective conservation and management actions.

## Supporting Information

Figure S1 **Frequency distribution of the 999 AUC values of the randomly drawn null-models (grey column) and the AUC value of ecological niche modeling (ENM) based on all presence data (dashed line).** The ENM AUC value is higher than its corresponding AUC value of the fitted null-model (*p*<0.01).
(TIF)

Figure S2 **Comparison values of bioclimatic variables in all known presence records between current and future climate scenarios.** The black solid horizontal line represents the median, the square symbol represents the mean, edges of box are quartiles, whiskers are 1th and 99th percentiles and black short lines are minimum and maximum.
(TIF)

Table S1 **Locations and coordinates of field presence records of the white-eared night heron** ***Gorsachius magnificus*** **.**
(DOC)

Table S2 **Explanatory environmental variables initially compiled for ecological niche modeling of** ***Gorsachius magnificus*** **.**
(DOC)

Table S3 **Contributions of specific environmental variables to the Maxent model. Values shown are averages over replicate runs.**
(DOC)
