# Supplementary figures and images for: Unveiling the Conservation Biogeography of a Data-Deficient Endangered Bird Species under Climate Change
Source: PLoS One. 2014 Jan 3;9(1):e84529. doi: 10.1371/journal.pone.0084529 (PMC3880300; doi:10.1371/journal.pone.0084529)

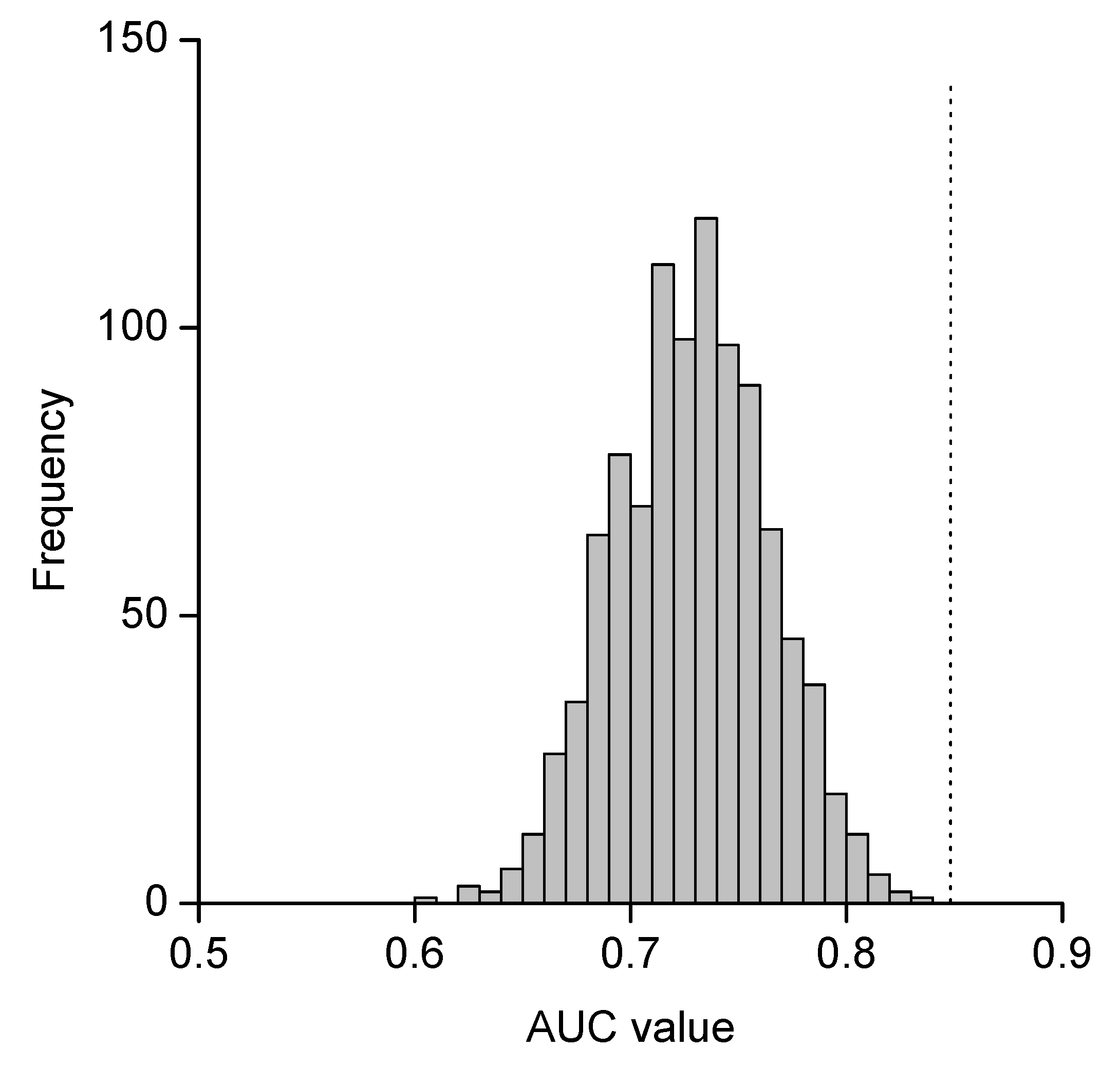

Supplement: Figure S1 — Frequency distribution of the 999 AUC values of the randomly drawn null-models (grey column) and the AUC value of ecological niche modeling (ENM) based on all presence data (dashed line). The ENM AUC value is higher than its corresponding AUC value of the fitted null-model (p<0.01). (TIF) [file pone.0084529.s001.tif]

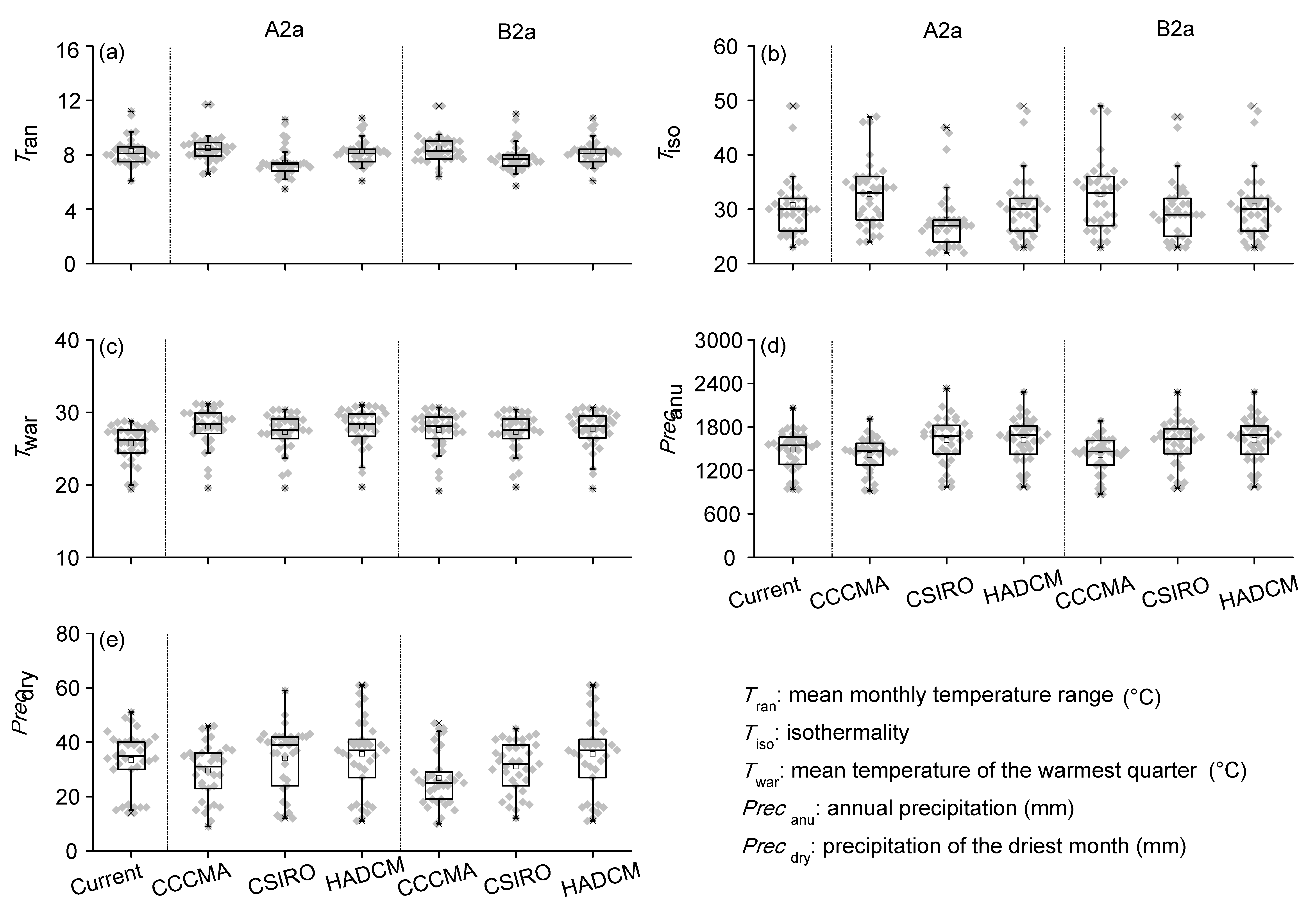

Supplement: Figure S2 — Comparison values of bioclimatic variables in all known presence records between current and future climate scenarios. The black solid horizontal line represents the median, the square symbol represents the mean, edges of box are quartiles, whiskers are 1th and 99th percentiles and black short lines are minimum and maximum. (TIF) [file pone.0084529.s002.tif]
