# Supplementary material for: Unveiling the Conservation Biogeography of a Data-Deficient Endangered Bird Species under Climate Change
Source: PLoS One. 2014 Jan 3;9(1):e84529. doi: 10.1371/journal.pone.0084529 (PMC3880300; doi:10.1371/journal.pone.0084529)
Supplement: Table S1 — Locations and coordinates of field presence records of the white-eared night heron Gorsachius magnificus . (DOC) [file pone.0084529.s003.doc]

**Table S1**

| Occurrence site | State/Province | Locality | Longitude | Latitude | Source |
| --- | --- | --- | --- | --- | --- |
| 1 | Anhui, CHN | Pengjialing, Qianshan Co. | 116.07 | 31.17 | He et al. 2007 |
| 2 | Anhui, CHN | Jingyang, Jingde Co. | 118.53 | 30.29 | He et al. 2007 |
| 3 | Anhui, CHN | Alligator protection center, Xuancheng City | 118.78 | 30.90 | He et al. 2007 |
| 4 | Fujian, CHN | Gutian | 118.74 | 26.58 | He et al. 2011 |
| 5 | Fujian, CHN | Jiangkou, Putian City | 119.18 | 25.53 | BirdLife International, 2012 |
| 6 | Guangdong, CHN | Huashui mountain, Yingde Co. | 112.54 | 24.44 | Gao et al. 2000 |
| 7 | Guangdong, CHN | Nanling NR. | 113.05 | 24.71 | Web source ^1^ |
| 8 | Guangdong, CHN | Ruyuan Co. | 113.30 | 24.68 | He et al. 2011 |
| 9 | Guangdong, CHN | Mashi, Shixing Co. | 114.00 | 25.00 | Gao et al. 2000 |
| 10 | Guangdong, CHN | Taiping, Shixing Co. | 114.07 | 24.97 | Gao et al. 2000 |
| 11 | Guangdong, CHN | Nankun Mountain, Longmen Co. | 114.08 | 23.75 | He et al. 2007 |
| 12 | Guangdong, CHN | Chebaling, Shixing Co. | 114.09 | 24.73 | BirdLife International, 2012 |
| 13 | Guangdong, CHN | Jiangkou, Shixing Co. | 114.17 | 25.00 | Gao et al. 2000 |
| 14 | Guangdong, CHN | Chebaling, Shixing Co. | 114.25 | 24.73 | BirdLife International, 2012 |
| 15 | Guangxi, CHN | Pingshan, Long’an Co. | 107.60 | 22.93 | BirdLife International, 2012 |
| 16 | Guangxi, CHN | Nanhuang, Fusui Co. | 107.90 | 22.35 | BirdLife International, 2012 |
| 17 | Guangxi, CHN | Fentinghe reservoir, Shangsi Co. | 108.24 | 22.28 | Web source ^2^ |
| 18 | Guangxi, CHN | Laohuling reservoir, Nanning City | 108.38 | 22.90 | BirdLife International, 2012 |
| 19 | Guangxi, CHN | Jiajiang reservoir, Heng Co. | 108.82 | 22.89 | BirdLife International, 2012 |
| 20 | Guangxi, CHN | Anhuai, Pingnan Co. | 110.45 | 23.63 | Web source ^3^ |
| 21 | Guizhou, CHN | Fangxiang, Leigong Mountain NR. | 108.28 | 26.44 | Li et al. 2008 |
| 22 | Hainan, CHN | Tianchi, Jianfengling, Ledong Co. | 108.86 | 18.75 | BirdLife International, 2012 |
| 23 | Hubei, CHN | Houhe NR.,Wufeng Co. | 110.58 | 30.10 | He et al. 2007 |
| 24 | Hubei, CHN | Wushanhu reservoir, Shennongjia | 110.79 | 31.70 | Zhou & Lu 2002 |
| 25 | Hubei, CHN | Yutauhe reservoir, Shennongjia | 110.80 | 31.72 | He et al. 2007 |
| 26 | Hubei, CHN | Taijitou, a bank of the Nanhe | 110.86 | 31.74 | Zhou & Lu 2002 |
| 27 | Hubei, CHN | Zhouwan, Maqiao town, Baokang Co. | 110.93 | 31.76 | Zhou & Lu 2002 |
| 28 | Hunan, CHN | Kangling NR.,Zhongfang Co. | 110.13 | 27.49 | He et al. 2007 |
| 29 | Hunan, CHN | Daoyuan reservoir, Liuyang Co. | 113.66 | 28.28 | He et al. 2007 |
| 30 | Jiangxi, CHN | Jiulianshan NR.,Longnan Co. | 114.45 | 24.59 | He et al. 2007 |
| 31 | Jiangxi, CHN | Jialu, Wuyuan Co. | 117.67 | 29.37 | He et al. 2007 |
| 32 | Jiangxi, CHN | Longjiang river, Jinggangshan City | 114.15 | 26.55 | He et al. 2011 |
| 33 | Yunnan, CHN | Fenghuang mountain, Nanjian Co. | 100.33 | 24.90 | He et al. 2007 |
| 34 | Yunnan, CHN | Ailao mountain, Xinping Co. | 101.50 | 23.95 | He et al. 2007 |
| 35 | Zhejiang, CHN | Tianmushan, Lin'an City | 119.43 | 30.34 | BirdLife International, 2012 |
| 36 | BacKan, VIE | Lung Ly, Xuan Lac District | 105.50 | 21.13 | Pilgrim et al. 2009 |

Web source: 1, <http://www.xinhuanet.com/chinanews/2007-10/10/content_11356754.htm>; 2, <http://www.wwfchina.org/bbs/bottomtest.shtm?channelid=7&ddd=292152&id=292152>; 3, <http://news.sina.com.cn/s/2007-10-01/151312667645s.shtml>. CHN, China; VIE, Vietnam.
