# Supplementary material for: Unveiling the Conservation Biogeography of a Data-Deficient Endangered Bird Species under Climate Change
Source: PLoS One. 2014 Jan 3;9(1):e84529. doi: 10.1371/journal.pone.0084529 (PMC3880300; doi:10.1371/journal.pone.0084529)
Supplement: Table S2 — Explanatory environmental variables initially compiled for ecological niche modeling of Gorsachius magnificus . (DOC) [file pone.0084529.s004.doc]

**Table S2**

| Code | Description | Source |
| --- | --- | --- |
| 1 | Annual Mean Temperature | http://www.worldclim.org, WorldClim 2004 |
| 2 | Mean monthly temperature range | http://www.worldclim.org, WorldClim 2004 |
| 3 | Isothermality (mean monthly temperature range /temperature annual range)(* 100) | http://www.worldclim.org, WorldClim 2004 |
| 4 | Temperature Seasonality (standard deviation * 100) | http://www.worldclim.org, WorldClim 2004 |
| 5 | Max Temperature of the Warmest Month | http://www.worldclim.org, WorldClim 2004 |
| 6 | Min Temperature of the Coldest Month | http://www.worldclim.org, WorldClim 2004 |
| 7 | Temperature Annual Range (BIO5 - BIO6) | http://www.worldclim.org, WorldClim 2004 |
| 8 | Mean Temperature of the Wettest Quarter | http://www.worldclim.org, WorldClim 2004 |
| 9 | Mean Temperature of the Driest Quarter | http://www.worldclim.org, WorldClim 2004 |
| 10 | Mean temperature of the warmest quarter | http://www.worldclim.org, WorldClim 2004 |
| 11 | Mean Temperature of the Coldest Quarter | http://www.worldclim.org, WorldClim 2004 |
| 12 | Annual precipitation | http://www.worldclim.org, WorldClim 2004 |
| 13 | Precipitation of the Wettest Month | http://www.worldclim.org, WorldClim 2004 |
| 14 | Precipitation of the driest month | http://www.worldclim.org, WorldClim 2004 |
| 15 | Precipitation Seasonality (Coefficient of Variation) | http://www.worldclim.org, WorldClim 2004 |
| 16 | Precipitation of the Wettest Quarter | http://www.worldclim.org, WorldClim 2004 |
| 17 | Precipitation of the Driest Quarter | http://www.worldclim.org, WorldClim 2004 |
| 18 | Precipitation of the Warmest Quarter | http://www.worldclim.org, WorldClim 2004 |
| 19 | Precipitation of the Coldest Quarter | http://www.worldclim.org, WorldClim 2004 |
| 20 | Growing degree days | http://www.sage.wisc.edu/atlas/index.php |
| 21 | Net primary productivity | http://www.sage.wisc.edu/atlas/index.php |
| 22 | Soil moisture | http://www.sage.wisc.edu/atlas/index.php |
| 23 | Soil pH | http://www.sage.wisc.edu/atlas/index.php |
| 24 | Soil organic carbon | http://www.sage.wisc.edu/atlas/index.php |
| 25 | Annual actual evapotranspiration | http://www.cgiar-csi.org |
| 26 | Annual potential evapotranspiration | http://www.cgiar-csi.org |
| 27 | Annual aridity index | http://www.cgiar-csi.org |
| 28 | Distance to water layer | Global Lakes and Wetlands Database |
| 29 | Normalized difference vegetation index, the average of values for 12 months over an 18-year period from 1982 to 2000 | http://edit.csic.es/Soil-Vegetation-LandCover.html |
| 30 | Human footprint index, an estimate of human influence based on human settlement, land transformation, accessibility and infrastructure data | http://www.ciesin.columbia.edu/wild_areas/, Last of the Wild Data Version 2, 2005 |
| 31 | Compound topographic index (CTI,commonly referred to as the wetness index) | http://edcdaac.usgs.gov/gtopo30/hydro/ |
