# Supplementary material for: Unveiling the Conservation Biogeography of a Data-Deficient Endangered Bird Species under Climate Change
Source: PLoS One. 2014 Jan 3;9(1):e84529. doi: 10.1371/journal.pone.0084529 (PMC3880300; doi:10.1371/journal.pone.0084529)
Supplement: Table S3 — Contributions of specific environmental variables to the Maxent model. Values shown are averages over replicate runs. (DOC) [file pone.0084529.s005.doc]

Table s3

| **Variable** | **Percent contribution** |
| --- | --- |
| Precipitation of the driest month (*Prec*_dry_) | 47.7 |
| Normalized difference vegetation index (NDVI) | 12.8 |
| Isothermality (*T*_iso_) | 12.6 |
| Distance to water layer | 11.2 |
| Human footprint index (HF) | 5.0 |
| Compound topographic index (CTI) | 3.1 |
| Mean temperature of the warmest quarter (*T*_war_) | 2.8 |
| Annual actual evapotranspiration (*AET*_anu_), | 2.4 |
| Annual precipitation (*Prec*_anu_) | 1.9 |
| Mean monthly temperature range (*T*_ran_) | 0.5 |
